# Dietary Advanced Glycation End Products in an Elderly Population with Diabetic Nephropathy: An Exploratory Investigation

**DOI:** 10.3390/nu14091818

**Published:** 2022-04-27

**Authors:** Mieke Steenbeke, Ignace De Decker, Sophie Marchand, Griet Glorieux, Wim Van Biesen, Bruno Lapauw, Joris R. Delanghe, Marijn M. Speeckaert

**Affiliations:** 1 Nephrology Unit, Department of Internal Medicine and Pediatrics, Ghent University Hospital, 9000 Ghent, Belgium; mieke.steenbeke@uzgent.be (M.S.); griet.glorieux@ugent.be (G.G.); wim.vanbiesen@ugent.be (W.V.B.); 2 Burn Center, Ghent University Hospital, 9000 Ghent, Belgium; ignace.dedecker@ugent.be; 3 Center for Healthy Food and Dietetics, Ghent University Hospital, 9000 Ghent, Belgium; sophie.marchand@uzgent.be; 4 Endocrinology Unit, Department of Internal Medicine and Pediatrics, Ghent University Hospital, 9000 Ghent, Belgium; bruno.lapauw@ugent.be; 5 Department of Diagnostic Sciences, Ghent University, 9000 Ghent, Belgium; joris.delanghe@ugent.be; 6 Research Foundation Flanders, 1000 Brussels, Belgium

**Keywords:** dietary advanced glycation end products (dAGEs), diabetic kidney disease (DKD), skin autofluorescence (SAF), urine spectroscopy

## Abstract

Advanced glycation end products (AGEs) are important in pathophysiology of type 2 diabetes mellitus (T2DM) and diabetic kidney disease (DKD). Dietary AGEs (dAGEs) contribute to the overall AGE pool in the body. Forty elderly T2DM patients with DKD were randomly allocated to a low-AGE (*n* = 20) or regular diabetic (*n* = 20) diet group. A three-day meal questionnaire was used to estimate average quantity of dAGEs. AGE accumulation was measured using skin autofluorescence and urine spectroscopy. sRAGE (soluble receptor AGE) was quantified using ELISA. After 8 weeks, the mean consumption of dAGEs was considerably reduced, both in the low-AGE diet (*p* = 0.004) and the control (*p* = 0.019) group. The expected urinary emission peak at 490 nm was shifted to 520 nm in some spectra. dAGEs did not correspond with urine AGE output. An AGE-limited diet for two months did not affect AGE content in skin and urine, or sRAGE concentration in the blood. The role of glycemia is likely to be greater than the impact of dAGE consumption. The unique observation of a fluorescence pattern at 520 nm warrants further examination, since it might point to genetic differences in AGE regulation, which could have clinical consequences, as AGE content depends on its formation and elimination.

## 1. Introduction

As a consequence of high-temperature cooking or processing of meals, such as baking, frying, or grilling, a simple yet ubiquitous reaction known as the Maillard reaction occurs both in vivo and ex vivo. Glycation of proteins is a post-translational modification that results in the formation of transient adducts that, with subsequent rearrangement and crosslinking, creating permanent residues known as advanced glycation end products (AGEs) [1]. Increasing evidence in the literature suggests an important role for AGEs in the generation of a state of increased oxidative stress and chronic subclinical inflammation, which underlies most modern chronic diseases [2]. Aside from their participation in the aging process, AGEs have been shown to play an important role in diabetes mellitus (DM), chronic kidney disease (CKD), cardiovascular disease (CVD), cataract, and Alzheimer’s disease [1,3]. The accumulation of AGEs in the kidneys and other tissues of diabetic patients has been linked to the development of diabetic nephropathy and vasculopathy [4]. In diabetic kidney disease and the ageing kidney, the accumulation of the AGEs-mediated receptor for AGEs (RAGE) causes oxidative stress and inflammation [5]. Two circulating soluble RAGE (sRAGE) isoforms without transmembrane and cytoplasmic domains, endogenous secretory RAGE (esRAGE) and cleaved RAGE (cRAGE), have been found in addition to the membrane-bound form of RAGE. The ligand-binding site in sRAGE might be used as a decoy receptor, which has an anti-atherosclerotic effect [6].

Current dietary recommendations for chronic disease prevention from the World Health Organization advise decreasing free sugars, saturated fat, and salt consumption [7]. Dietary AGEs (dAGEs) are contributors to the total body AGE pool [8] and increase CVD markers in patients with DM [9]. The stressful and continuous workaholic atmosphere has steered individuals towards pre-prepared and processed foods, which, although delectable to the palate, possess the highest amount of exogenous AGEs compared to freshly home-cooked meals. The majority of dAGEs are produced in foods prepared using dry heat technology [1,3]. AGEs have been found in a wide variety of food products in the Western diet, including biscuits, bread, cheese, peanut butter, and processed meats, as well as carbonated drinks containing high fructose corn syrup, such as cola, and some fermented products, such as wine and beer [1]. dAGEs have a poor biological availability; on average, 10–30% of ingested AGEs are absorbed into the systemic circulation. Only 30% of ingested AGEs are eliminated in the urine of people with normal kidney function during the next 48 h, and this number drops proportionally to as low as 5% in people with severe kidney dysfunction [8].

Changing the cooking process from high dry heat to moderate heat and high humidity can reduce dAGEs regardless of nutritional content [2]. As limiting dAGEs is a straightforward, safe, effective, and realistic approach of reducing toxic AGE excess and maybe cardiovascular related damage [10], it may be considered as a preventive measure. Several clinical trials have been conducted in a variety of conditions over the last decade (in CKD patients without DM, diabetic patients without CKD, and diabetic patients with CKD while maintaining the same dAGEs intake, but receiving an oral AGE-binder) demonstrating that the application of dAGEs reduction reduces not only the systemic levels of AGEs, but also the levels of markers of oxidative stress and inflammation [2,3].

In this pilot study, we sought to investigate the effect of a low-AGE diet for 8 weeks on the presence of AGEs in patients with diabetic kidney disease (DKD) using two distinct approaches to measure AGE content, namely skin autofluorescence (SAF) and urine spectroscopy.

## 2. Materials and Methods

### 2.1. Study Participants

In this intervention study, we looked at the effect of dietary education on dAGEs with the goal of minimizing AGE intake. Personal data such as age, gender, dominant arm side, and smoking history were obtained from each patient. Forty diabetic kidney disease (DKD) patients (CKD stages 3–4) were randomly assigned to one of two groups: low-AGE or regular diabetic diet. A number of about 9 patients was calculated for each group, to obtain a power (1 − β) of 0.80 and an α-error of 0.05 [11], taking into account a change of approximately 7% in subjects reducing dAGEs intake, and AGEs in circulation beginning at levels of approximately 9900 ± 0.5 AU [12,13]. 

In the treatment group of 20 patients, information about dAGEs and the cooking method was provided by a dietician of the Ghent University Hospital. At the baseline appointment, the dietician explained how the patient may easily reduce dAGE consumption by simply switching from a high dry heat treatment to a low heat and high humidity application, regardless of its nutritional content. A large database containing the dAGE content of typical meals was utilized to estimate daily dAGE consumption as well as to provide tailored advice on how to minimize the AGE-load in the human body [3,12]. This information was not supplied in the control group, which included the remaining 20 patients on a standard diabetic diet. The average amount of dAGE was determined using a three-day food questionnaire designed to get an approximation of the normal daily diet at baseline and after 8 weeks. All foods and beverages were meticulously documented every 24 h for three days. This covered the brand, the amount, the cooking methods, and the fat products utilized. The mean quantity of dAGE was computed for each period and patient [3,14]. Standardized food models and a food photo book (Portiegroottes boek, Valetudo Consulting, fourth edition, October 2017) were used to improve the accuracy of portion size estimate.

The approval of this study was granted by the Ethical committee of the Ghent University Hospital (BC-08494, dd 15 February 2021). Informed consent was obtained from all participants before the start of the study.

### 2.2. Routine Laboratory Parameters

Blood and first morning urine collection were performed at baseline and after 8 weeks. The routine laboratory parameters were carried out in the routine laboratory of Ghent University Hospital, Belgium. Hemoglobin A1c (HbA1c) in ethylenediamine tetraacetic acid (EDTA) plasma was analyzed by ion exchange chromatography on the Tosoh HLV-723 G8 (Tosoh, Tokyo, Japan), which also included measurement of the labile fraction of HbA1c. Labile HbA1c is a Schiff base formed during non-enzymatic glycation of hemoglobin, and its concentration varies with acute changes in plasma glucose level [15]. Serum and urinary creatinine were determined by a photometric isotope dilution mass spectrometry (ID-MS) calibrated alkaline picrate method using the Architect c16000 (Abbott Laboratories, Chicago, IL, USA). The estimated glomerular filtration rate (eGFR) was calculated with the chronic kidney disease epidemiology collaboration (CKD-EPI) formula [16]. The urinary albumin concentration was determined on a Behring Nephelometer analyzer II (Siemens, Marburg, Germany) by immunonephelometry.

### 2.3. Measurement of AGEs and sRAGE

AGE accumulation was measured using SAF and urine spectroscopy. Excitation-emission spectra of the fasting urine samples were recorded using a Flame miniature spectrometer (FLAME-S-VIS-NIR-ES, 350–1000 nm, Ocean Optics, Dunedin, FL, USA), equipped with a high-power LED light source (365 nm, Ocean Optics) and reflection probe (QR400-7-VIS-BX, Ocean Optics). One mL urine was transferred into a quartz cuvette with a 1 cm path length (Carl Zeiss, Oberkochen, Germany). The reflection probe was positioned against the cuvette, and a black background was used. Measurements were averaged over 128 scans. Using an excitation wavelength of 365 nm, the fluorescence spectra of urinary AGEs were recorded at a 400–620 nm emission range. After background correction, the fluorescence signal of each urine sample was measured. Normalized fluorescence spectra were prepared by dividing the relative fluorescence intensity at each wavelength by the (maximum) relative fluorescence intensity at the (corresponding) peak wavelength. As the urinary concentration of AGEs depends on the 24 h urine volume, the relative fluorescence intensity (expressed in arbitrary units (AU)) was adjusted for the urinary creatinine concentration. The mean autofluorescence (AF) value (*n* = 3) at 440, and 490 or 520 nm, and the sum of the three autofluorescence values of the corrected spectra were used.

Since fluorescence spectroscopy is a valuable and commonly employed method for the detection and measurement of autofluorescent AGEs [17,18], Maillard-type AF measurements (excitation 365 nm, emission 400–620 nm) on the skin were performed using the same method as described above. To have a fixed measurement position on the skin, a Tegaderm Film (3 M, Diegem, Belgium) in the middle a cut out of a 4 cm^2^ square was placed on the volar side of the dominant and non-dominant side of the arm at elbow height of each patient. The probe was placed in the open square of the Tegaderm Film, and thus directly on the skin. The measurement was repeated 3 times on each side on a different position in the open square. SAF1 was calculated by dividing the average light intensity emitted per nm for the 407–677 nm range by the average light intensity per nm over the 342–407 nm range. The mean of the three measurements was taken.

Next to using the Flame miniature spectrometer (Ocean Optics), the amount of AGE accumulation in the skin was also estimated using an AGE-reader (DiagnOptics, Groningen, the Netherlands). SAF was measured on the volar side of the forearm, approximately 10 cm below the elbow, both at the dominant and non-dominant side. The AGE-reader illuminates at a skin surface of 4 cm^2^ with ultraviolet light wavelengths between 300–420 nm (peak intensity 370 nm). The emitted and reflected light intensities were measured with a spectrometer in the range of 300–600 nm. The ratio of the average intensity of the emitted light (wavelengths between 420 nm to 600 nm) divided by the average intensity of reflected light (wavelengths between 300–420 nm), multiplied by 100 was measured as SAF2 and expressed in AU. An average of three measurements was taken. In patients with diabetes, SAF2 has an intra-individual, intra-day variability of 4.2–5.0% and a seasonal variability of 5.9% [19].

Quantitative determination of sRAGE in EDTA plasma was determined by a commercial enzyme-linked immunosorbent assay (ELISA) (DY1145, R&D Systems, Minneapolis, MN, USA).

### 2.4. Statistical Analysis

All data analysis was performed with MedCalc version 9.4.2.0 (MedCalc Software, Ostend, Belgium). Normality of distributions was assessed using the Shapiro–Wilk test. In the case of normal distribution (if necessary using a log-transformation), data were presented as mean ± standard deviation. The median with corresponding interquartile range (IQR) were determined for not normally distributed data. To investigate correlations, the Pearson’s correlation coefficient (r) or Spearman’s coefficient of rank correlation (ρ) was calculated. To compare two groups with normal distribution, the paired samples *t*-test and independent samples t-test were used. To compare two groups without a normal distribution, the Wilcoxon test (paired samples) and the Mann-Withney U test (independent samples) were used.

Multiple regression was carried out to study the confounders of AGEs present in the human body. A *p*-value < 0.05 was considered a priori to be statistically significant.

## 3. Results

### 3.1. Study Participants

Forty DKD patients were randomly allocated to the control group (*n* = 20, 16 men, 4 females, age: 73.9 ± 6.9 years, 2/20 smoking, baseline serum creatinine: 1.78 ± 0.59 mg/dL and eGFR: 39 ± 12 mL/min/1.73 m^2^) and treatment group (*n* = 20, 13 males, 7 females, age: 74.0 ± 4.2 years, 1/20 smoking, baseline serum creatinine: 1.69 ± 0.41 mg/dL and eGFR: 38 ± 10 mL/min/1.73 m^2^).

### 3.2. Urine Samples

**Figure 1:**
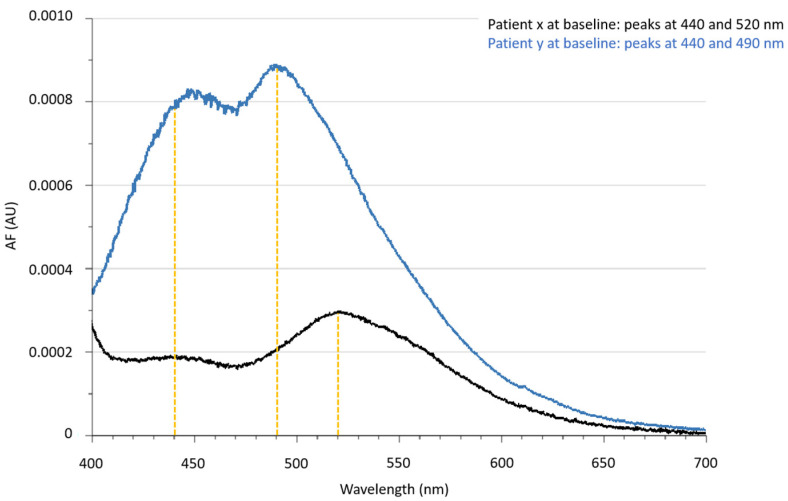
Corrected urinary autofluorescence spectra of two patients at baseline to point out the different emission spectra with maximum peaks in the emission range at 440, 490 and 520 nm. Abbreviations: AF: autofluorescence, AU: arbitrary units.

Surprisingly, two types of spectra could be separated in both groups: whereas the peak at 440 nm was found in all participants at both time points, at baseline 30 of 40 patients had a peak at 490 nm, and thus the peak at 520 nm was only observed in 10 of 40 patients, and after 8 weeks, 33 of 40 patients had a peak at 490 nm, and thus the peak at 520 nm was only observed in 7 of 40 patients (Figure 1). This phenomena, with a peak at 520 nm, was seen in six patients at both time points.

No significant variations in AGE content through urinary AF_440nm_ and subgroups AF_490nm_ and AF_520nm_ were found over time in the control and treatment groups, nor through the sum of the three urinary autofluorescence values (AF_∑440+490+520nm_). There were no significant changes in AGE content between the control and treatment groups as measured in the urine neither at baseline nor after 8 weeks (Table 1).

No correlation was observed between urine albumin-creatinine ratio (uACR), and AF_440nm_, AF_490nm_, AF_520nm_, and AF_∑440+490+520nm_ (*n* = 75, ρ = −0.008, *p* = 0.944; *n* = 59, ρ = −0.004, *p* = 0.975; *n* = 16, ρ = −0.063, *p* = 0.816; *n* = 75, ρ = 0.013, *p* = 0.914).

**Table 1:** Blood, skin and urinary parameters, and dietary advanced glycation end products (dAGEs) in the control and treatment group over a period of 8 weeks.

			Control Group (*n* = 20)			Treatment Group (*n* = 20)				
			Baseline	8 Weeks	*p* Value vs. Baseline	Baseline		8 Weeks		
							*p* Value vs. the Control Group		*p* Value vs. the Control Group	*p* Value vs. Baseline
Blood		Creatinine (mg/dL)	1.78 ± 0.59			1.69 ± 0.41				
		eGFR (mL/min/1.73 m^2^)	39 ± 12			38 ± 10				
		HbA1c (mmol/mol)	60.7 ± 12.4	59.8 ± 12.3	0.369	57.4 ± 9.3	0.353	56.2 ±7.1	0.265	0.327
		labile HbA1c (%)	3.2 ± 0.6	3.1 ± 0.5	0.224	2.9 ± 0.5	0.140	2.9 ± 0.3	0.349	0.947
		sRAGE (pg/mL)	441.1 ± 130.2	448.0 ± 177.3	0.753	556.7 ± 167.0	0.019	560.5 ± 219.0	0.082	0.866
Skin	Dominant	SAF_1_ (AU)	0.037 ± 0.008	0.039 ± 0.008	0.133	0.041 ± 0.012	0.186	0.045 ± 0.014	0.147	0.069
		SAF_2_ (AU)	3.3 ± 0.6	3.4 ± 0.7	0.132	3.4 ± 0.6	0.451	3.3 ± 0.5	0.516	0.088
	Non-dominant	SAF_1_ (AU)	0.041 ± 0.008	0.039 ± 0.010	0.401	0.042 ± 0.011	0.654	0.046 ± 0.015	0.076	0.104
		SAF_2_ (AU)	3.3 ± 0.6	3.5 ± 0.8	0.030	3.4 ± 0.7	0.603	3.4 ± 0.7	0.610	0.671
Urine	uACR (mg albumin/mmol creatinine)		5.4 (2.1–86.0)	3.7 (1.5–51.6)		2.4 (1.0–7.7)		3.6 (1.0–6.2)		
	AF_440nm_ (AU)		5.8 (3.9–9.9)	5.7 (4.4–7.6)	0.235	7.1 (5.6–8.6)	0.507	6.7 (4.7–9.6)	0.262	0.701
	AF_490nm_ (AU)		5.4 (4.2–9.7) (*n* = 16) ^+^	6.4 (4.9–8.7) (*n* = 17) ^+^	0.774	8.4 (6.9–9.0) (*n* = 14) ^+^	0.190	7.9 (6.2–12.1) (*n* = 16) ^+^	0.130	0.354
	AF_520nm_ (AU)		11.3 ± 5.0 (*n* = 4) ^+^	8.3 ± 4.3 (*n* = 3) ^+^	0.111	9.5 ± 6.1 (*n* = 6) ^+^	0.637	6.0 ± 3.3 (*n* = 4) ^+^	0.451	0.503
	AF_∑440+490+520nm_ (AU)		12.7 (8.2–20.9)	12.2 (9.6–16.3)	0.276	16.2 (12.4–18.1)	0.409	14.6 (10.3–21.3)	0.317	0.648
Food record	dAGEs (MU AGEs/day)		15.5 (12.5–21.9)	12.8 (8.7–16.0)	0.019	14.2 (11.3–19.4)	0.536	11.6 (9.4–13.0)	0.636	0.004

Data are presented as mean ± standard deviation (SD) or as median with interquartile range (IQR). ^+^ two subgroups with a peak at 490 or 520 nm were obtained. Abbreviations: AF: autofluorescence; AU: arbitrary units; dAGEs: dietary advanced glycation end products; FLAME: Flame miniature spectrometer; HbA1c: hemoglobin A1c; IQR: interquartile range; MU: million units; SAF: skin autofluorescence; SD: standard deviation; sRAGE: soluble receptor for advanced glycation products; uACR: urine albumin–creatinine ratio; ∑: sum.

### 3.3. Skin

When comparing the AGE content in the skin (SAF_1_ and SAF_2_ results) over time, no significant differences were found for the control and treatment group. The SAF_2_ result of the control group at the non-dominant arm side (at baseline: 3.3 ± 0.6; after 8 weeks: 3.5 ± 0.8, *p* = 0.030) was the only significant difference found. No significant differences were found comparing the control and treatment group at baseline and after 8 weeks (Table 1).

### 3.4. Blood Samples

HbA1c and labile HbA1c were shown to have a substantial positive correlation at baseline and after 8 weeks in the entire study group (*n* = 40) (r = 0.621; *p* < 0.0001; r = 0.662; *p* < 0.0001). There were no significant changes in HbA1c and labile HbA1c over time in the control or treatment groups. There were no significant differences in HbA1c and labile HbA1c in the control and treatment groups at baseline or after 8 weeks, respectively (Table 1).

sRAGE plasma concentrations showed no significant difference over time in the control or treatment group. sRAGE concentration was significantly higher in the treatment group versus the control group at baseline (*p* < 0.019) and after 8 weeks (*p* = 0.082) (Table 1).

No associations were observed between sRAGE concentration, and SAF_1_ and SAF_2_ at the dominant (*n* = 79, ρ = 0.092, *p* = 0.421; *n* = 80, ρ = 0.105, *p* = 0.354) and non-dominant side (*n* = 77, ρ = 0.145, *p* = 0.207; *n* = 78, ρ = 0.175, *p* = 0.125).

### 3.5. Three Day Food Questionnaires

When comparing the dAGEs intake over time in the control group (at baseline: 15.5 (12.5–21.9) MU AGEs/day; after 8 weeks: 13.1 ± 5.1 MU AGEs/day, *p* = 0.019) and treatment group (at baseline: 14.2 (11.3–19.4) MU AGEs/day; after 8 weeks: 11.6 (9.4–13.0) MU AGEs/day, *p* = 0.004) a significant decrease was found in both groups (Table 1). dAGEs intake was comparable in the control and treatment group at baseline and after 8 weeks. No association was found between AF_440nm_, AF_490nm_, AF_520nm_, AF_∑440+490+520nm_, and dAGEs per day (*n* = 79, ρ = −0.117, *p* = 0.305; *n* = 62, ρ = −0.157, *p* = 0.223; *n* = 17, ρ = 0.076, *p* = 0.772; *n* = 79, ρ = −0.010, *p* = 0.382).

### 3.6. Multiple Regression

No significant relations between confounders (HbA1c, labile HbA1c, sRAGE, and uACR) of AGEs present in the human body were found.

## 4. Discussion

Following 8 weeks of an AGE-lowering diet, no significant reduction in AGE content and sRAGE concentration was found in the treatment group. Similarly, no significant variations in practically all laboratory data were identified throughout the study period. This negative finding suggests that additionally lowering the intake of dAGEs with the aim to lower AGE burden in elderly type 2 diabetes mellitus (T2DM) patients with DKD may be challenging. Of note, dietary advice is already part of their treatment, which accounts for a baseline reduction in AGEs.

The estimation of dAGEs is prone to inaccuracies. Because the quantity of dAGEs included in meat and fats is crucial to the mean amount of estimated dAGEs, the assessment of the serving portion might have an effect on the number of dAGEs present. Although a comprehensive list of the amounts of dAGEs [3,14] in various meals and beverages is provided, a number of goods and the influence of appropriate cooking procedures are not included. Only the quantity of dAGEs in blond beer (specifically Budweiser) and toasted bread is reported, whereas the degree of roasting is not considered. The quantity of dAGEs found in baguette, Belgian biscuits, Belgian cheese, and other fat items has not been specified. The majority of AGEs are derived from the consumption of meat and fat products (namely butter and margarine); nevertheless, the dependability of the estimates of the utilized fat products is called into doubt. Acids (specifically, acetic acid, lemon and lime juice, and red wine) can be utilized to prevent the production of dAGEs [3]. This may be accomplished by marinating meat or fish in acid prior to baking. Unfortunately, it is unclear how to account for this effect on the quantity of dAGEs present. Despite agreements, the dAGEs computations were carried out by various people, which might have resulted in a divergent interpretation of the data. Furthermore, the elderly often do not consume fast foods, which are high in dAGEs. Furthermore, no standardized low-AGE meals were produced in this trial, which may have impacted the outcomes.

The AGE database [14] was created using an ELISA test, which may overestimate or underestimate the real AGE level of a dietary component since it has not been verified against liquid chromatography–mass spectrometry (LC-MS). There is an alternative LC-MS-based database [20] available, however it only comprises a limited number of items.

Only low molecular weight (LMW) free AGEs, including peptide-bound forms, and carbonyls may be quickly absorbed from the gut and contribute to the body burden of AGEs. In contrast, high molecular weight (HMW) protein-bound AGEs may be poorly absorbed due to inadequate breakdown by gastrointestinal enzymes [21]. Aside from dAGEs (which are poorly absorbed) [8,21], a large component of circulating AGEs comes from T2DM’s impaired glucose metabolism, which is not significantly affected by dAGE inflow. As a result of glycemic stress, patients with T2DM have considerably higher AGE levels than the general population [22]. The lack of association between dAGEs and urine fluorescence results indicates the difficulty in estimating the quantity of consumed AGEs (due in part to the influence of cooking on AGE formation) and the varied absorption of ingested AGEs. Few studies in humans have looked at the metabolic fate of dAGEs and the AGE content in urine and feces following AGE restriction in T2DM with CKD [9]. In a pilot study with peritoneal dialysis patients, dAGE restriction altered the bacterial gut microbiota [23]. The noteworthy variation in urine AGE spectrum demonstrates the interindividual heterogeneity of AGE metabolism: only 21% of the urinary fluorescence emission spectra of the examined group displayed a peak at 520 nm, which appeared to be constitutionally determined.

The present findings are in agreement with the study of Chilelli et al., who did not find a significant reduction in serum AGEs after a long follow-up of 12 weeks of CKD patients on a low-AGE diet [11]. Although animal studies [24,25,26] suggest that dAGEs may target pancreatic islets, compromising the function of insulin-secreting beta cells, the absence of effect of a reduced AGE diet on HbA1c and labile HbA1c is consistent with prior human research [27,28,29,30,31,32,33,34]. Patients with CKD, like those with diabetes, exhibit higher levels of oxidative stress, namely circulating 8-isoprostanes, a sign of lipid peroxidation [35]. A low-quality study provided indications that an AGE-restricted diet might reduce plasma 8-isoprostanes in CKD patients. However, because just a small number of individuals (*n* = 9) were included, the generalizability of these findings is restricted. Furthermore, the authors did not indicate whether these individuals had comorbidities such as diabetes [9].

A significant difference in concentration of sRAGE was only found at the beginning of the study between the control and treatment group. Although lowering the dAGEs in the treatment group, no significant decrease in concentration of sRAGE over time was noticed in this population. sRAGE is known to accumulate in diabetic patients with CKD due to increased production and decreased kidney filtration [36]. The latter can have a larger impact than lowering the dAGEs for 2 months. No associations between sRAGE concentration in the blood, and SAF_1_ and SAF_2_ at the dominant and non-dominant side was observed, as sRAGE in blood monitors the short-term effect, changes in SAF may be observed over a period of years.

Finally, medication intake in both the treatment and control groups could have influenced the reported results: for example, angiotensin converting enzyme inhibitors act as pre- and post-Amadori inhibitors as well as transition metal ion chelators, angiotensin receptor blockers are agonists of peroxisome proliferator-activated receptor gamma (PPAR), and statins stimulate shedding of the RAGE [37]. Intake of vitamin B6, a recognized AGE inhibitor, may further skew the outcome of the intervention [38]. Due to the small number of smoking patients (3/40), the effect of tobacco smoke as an additional source of exogenous AGEs is limited [39].

Between weeks 0 and 8, a significant difference in the control group was detected using the AGE-reader on the non-dominant side of the measurements. The SAF_2_ value was reduced by an average of 6%. Although no substantial difference over time was expected in the control group, this can be explained by the AGE reader’s variance, making the aforementioned finding negligible [19]. The advantages of a reduced AGE diet may emerge over years rather than weeks or months when AGEs accumulate, glycate other proteins, or are absorbed by tissues. As a result, future research should involve a longer follow-up time [40].

## 5. Conclusions

The present study does not suggest an impact of dAGE restriction on content to AGEs and sRAGE in elderly patients with T2DM. Although a low AGEs diet might decrease the risk associated with CKD, such as inflammation and oxidative stress, there is a lack of high quality randomized trials which have investigated this association. There is currently insufficient evidence to recommend dAGEs restriction in order to reduce glyco-oxidative stress in these patients [10,11,40,41]. The remarkable finding of a fluoresence pattern at 520 nm deserves further investigation, since it may point to genetic differences in AGE handling, which might have clinical consequences. The genetic ability to detoxifying mechanisms against the buildup of AGEs may impact variations in circulation and urine AGEs [42]. The amount of AGEs in the body is determined not only by the pace at which they are formed, but also by their capacity to be eliminated by intrinsic detoxifying mechanisms. Among the putative detoxifying strategies against AGEs are reduced glutathione, which catalyzes the conversion of methylglyoxal to the less hazardous D-lactate by glyoxalase I and II [43]. Other enzymatic systems include fructosamine kinases [44], which phosphorylate and destabilize Amadori products, causing them to spontaneously degrade.

## Data Availability

The data presented in this study are available in an anonymized format on request from the corresponding author.
